# High‐Valence Nickel Single‐Atom Catalysts Coordinated to Oxygen Sites for Extraordinarily Activating Oxygen Evolution Reaction

**DOI:** 10.1002/advs.201903089

**Published:** 2020-01-20

**Authors:** Yaguang Li, Zhong‐Shuai Wu, Pengfei Lu, Xiao Wang, Wei Liu, Zhibo Liu, Jingyuan Ma, Wencai Ren, Zheng Jiang, Xinhe Bao

**Affiliations:** ^1^ Dalian National Laboratory for Clean Energy Dalian Institute of Chemical Physics Chinese Academy of Sciences 457 Zhongshan Road Dalian 116023 China; ^2^ Hebei Key Lab of Optic‐electronic Information and Materials The College of Physics Science and Technology Hebei University Baoding 071002 China; ^3^ University of Chinese Academy of Sciences 19 A Yuquan Rd, Shijingshan District Beijing 100049 China; ^4^ Shenyang National Laboratory for Materials Science Institute of Metal Research Chinese Academy of Sciences Shenyang 110016 China; ^5^ Shanghai Institute of Applied Physics Chinese Academy of Sciences Shanghai 201204 China; ^6^ State Key Laboratory of Catalysis Dalian Institute of Chemical Physics Chinese Academy of Sciences 457 Zhongshan Road Dalian 116023 China

**Keywords:** high valence, nickel, oxygen coordination, oxygen evolution reaction, single‐atom catalysts

## Abstract

Single‐atom catalysts (SACs) are efficient for maximizing electrocatalytic activity, but have unsatisfactory activity for the oxygen evolution reaction (OER). Herein, the NaCl template synthesis of individual nickel (Ni) SACs is reported, bonded to oxygen sites on graphene‐like carbon (denoted as Ni‐O‐G SACs) with superior activity and stability for OER. A variety of characterizations unveil that the Ni‐O‐G SACs present 3D porous framework constructed by ultrathin graphene sheets, single Ni atoms, coordinating nickel atoms to oxygen. Consequently, the catalysts are active and robust for OER with extremely low overpotential of 224 mV at current density of 10 mA cm^−2^, 42 mV dec^−1^ Tafel slope, oxygen production turn over frequency of 1.44 S^−1^ at 300 mV, and long‐term durability without significant degradation for 50 h at exceptionally high current of 115 mA cm^−1^, outperforming the state‐of‐the‐art OER SACs. A theoretical simulation further reveals that the bonding between single nickel and oxygen sites results in the extraordinary boosting of OER performance of Ni‐O‐G SACs. Therefore, this work opens numerous opportunities for creating unconventional SACs via metal–oxygen bonding.

## Graphical abstract

Nickel single‐atom catalysts bonded to oxygen sites on graphene‐like carbon nanosheets are synthesized as extraordinarily active and durable electrocatalysts for the oxygen evolution reaction, showing the oxygen production turn over frequency of 1.44 S^−1^ at 300 mV, and low overpotential of 224 mV at current density of 10 mA cm^−2^.

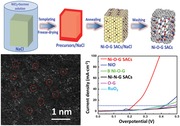

The ever‐increasing demands for clean energy and growing concerns on environmental issues have greatly accelerated the exploitation of molecular hydrogen (H_2_) that has been proposed as a future energy carrier.^1^ However, the energy conversion efficiency of hydrogen production by electrocatalytic water splitting is usually obstructed by the large overpotential of kinetically sluggish oxygen evolution reaction (OER).^2^ Therefore, highly active and stable catalysts are urgently required to boost OER efficiency by minimizing the overpotential.^3^ Although the noble metal oxides of IrO_2_ and RuO_2_ are the benchmark OER catalysts, their scarcity and high cost hinder the wide applications.^4^ Thus, non‐precious materials, such as transition metal complexes (e.g., Ni, Co, Mn, and Fe),^5^ oxides/hydroxides,^2^, ^6^ doped nanocarbons (e.g., N, O, S, and P),^7^ organic species,^8^ have been intensively researched as valuable electrocatalysts for OER. Despite their low overpotential, the active sites of these OER catalysts are normally existed in nanoparticle form, tend to be sparsely distributed at the primarily exposed facet or edge sites.^9^ As a result, the interior active sites of the catalysts cannot be fully utilized, eventually resulting in the waste of catalysts and partial loss of the entire electrocatalytic activity.^10^

Recently, single‐atom catalysts (SACs) incorporated into 2D substrates^11^ are becoming highly attractive for various reactions and systems, e.g., oxygen reduction reaction,^12^ hydrogen evolution reaction,^13^ CO_2_ reduction,^14^ CO oxidation,^11^ ethanol oxidation,^15^ nitroarenes hydrogenation,^16^ exhibiting nearly 100% atom utilization, high catalytic activity, long‐term stability, and exceptional selectivity. Special emphasis is given to the SACs for OER.^17^ To date, the reported SACs in electrocatalysis are generally bonded with nitrogen (N) coordinator, yielding unsatisfactory catalytic activity for OER.^18^ Since the chemical states of SACs are substantially regulated by the coordination number and spatial structure of coordinated elements,^14^, ^17^ therefore developing displacement of N with other atoms bond to SACs is promising to accelerate the electron transfer, modulate the adsorption of oxygen groups (e.g., H_2_O and OH*) and eventually improve the OER activity and durability.^9^, ^19^, ^20^ Among the coordinated elements, oxygen (O) is an ideal coordinator that can remarkably regulate the electronic properties of metal atoms due to its superior electronegativity.^21^ However, the M—O coordination is much weaker than M—N bond,^22^ leading to a challenge on the protection and stabilization of the oxygen bond metal sites in single‐atom state.^20^, ^23^ Therefore, the exploitation of SACs via M—O coordination in electrocatalysis has remained untouched so far.

Here, we report the first demonstration of nickel (Ni) SACs coordinated to oxygen sites on graphene‐like carbon (denoted as Ni‐O‐G SACs), which display 3D interconnected porous framework composed of ultrathin graphene‐like sheets, single atomic distribution of Ni sites, Ni—O configuration, and high valence state of single Ni atoms. As a result, the Ni‐O‐G SACs for OER in alkaline electrolyte possess a current density of 10 mA cm^−2^ at a very small overpotential of 224 mV, two times lower than its counterpart of NiO nanoparticles anchored on graphene (450 mV), superior to the start‐of‐the‐art SACs^24^, ^25^ and pure Ni‐based catalysts^26^, ^27^ and well comparable to the best OER catalysts so far.^28^, ^29^ Further, the Ni‐O‐G SACs showed a low 42 mV·dec^−1^ Tafel slope and outstanding long‐term stability. Density functional theory (DFT) calculations confirmed that the high binding energy of single Ni atoms lead to the efficient and durable OER performance of Ni‐O‐G SACs.

The schematic of the NaCl template synthesis of Ni‐O‐G SACs is illustrated in **Figure** **1**a. Sucrose (C_12_H_22_O_11_) contains only oxygen, carbon, and hydrogen elements, which can be used as the source to prepare Ni‐O‐G SACs totally without nitrogen incorporation. First, nickel chloride (NiCl_2_) and sucrose (C_12_H_22_O_11_) were dissolved in NaCl solution. In this stage, the Ni (II) ions will be spontaneously immobilized in single atomic state in solution. To stabilize the coordination of Ni (II) ions with sucrose in atomic dispersed state,^30^ a fast freeze‐drying technique was implemented to the mixture during drying process, thus avoiding the aggregation of Ni (II) ions. Meanwhile, ultrathin layer of sucrose was wrapped around the NaCl crystals. Afterwards, the freeze‐dried precursor was annealed at 700 °C in argon atmosphere to carbonized sucrose as graphene to generate 2D Ni‐O‐G nanosheets/NaCl. Note that, the optimization of annealing temperature is critical for the formation of mono‐dispersed Ni SACs (Figures S1 and S2, Supporting Information). Finally, Ni‐O‐G SACs were attained after removing NaCl templates (see details in Supporting Information).

**Figure 1:**
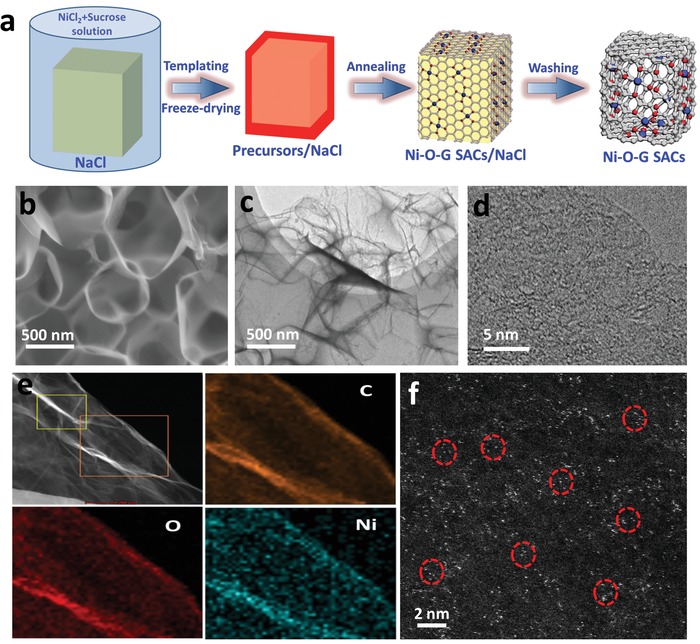
a) Schematic illustration of the synthesis of Ni‐O‐G SACs. b) SEM, c) TEM, d) HRTEM images, and e) STEM image and corresponding elemental mapping images of C, O, and Ni of Ni‐O‐G SACs. f) Atomic‐scale HAADF‐STEM image of Ni‐O‐G SACs. The single Ni atoms show bright dots marked with red circles.

Detailed structure and morphology of the Ni‐O‐G SACs (3.1 wt% Ni amount) were elucidated using a variety of characterization techniques. X‐ray diffraction (XRD) pattern of the Ni‐O‐G SACs presented two broad diffraction peaks at 23.2° and 43.6°, derived from the graphene frame. Besides this, no peaks of Ni metals, metal oxides, metal carbides were observed (Figure S3a, Supporting Information).^31^ X‐ray photoelectron spectroscopy (XPS) and Raman spectrum confirmed the existence of oxygen and defectization of graphene in this sample,^32^ indicating the incorporation of oxygen in graphene (Figure S3b, c, Supporting Information). Nitrogen adsorption and desorption isotherm of Ni‐O‐G SACs presented high specific surface area of 580 m^2^ g^−1^ (Figure S3d, Supporting Information). Scanning electron microscopy (SEM) and transmission electron microscopy (TEM, Figure 1b–d) images of Ni‐O‐G SACs showed the 3D interconnected macroporous framework constructed by 2D ultrathin graphene‐like nanosheets, implying the successful template replication of NaCl crystals. Therefore, the key role of NaCl crystals was served as the template platform for the creation of 3D hollow framework and the formation of 2D sheet structure of Ni‐O‐G SACs. Moreover, nanoparticles were absent in 2D Ni‐O‐G nanosheet, which is in good agreement with the XRD result. Based on the wrinkles and edges, the average thickness of 2D Ni‐O‐G nanosheet was approximately 2 nm (Figure S4, Supporting Information). The homogeneous dispersion of C, O, and Ni elements on Ni‐O‐G nanosheets was further verified by scanning transmission electron microscopy (STEM, Figure 1e). To identify the invisible Ni atoms of Ni‐O‐G sheets, the aberration‐corrected high‐angle annular dark‐field STEM (HAADF‐STEM) technique was used. Clearly, the atomic‐scale HAADF‐STEM image (Figure 1f) displayed several bright tiny dots, corresponding to heavy Ni atoms evenly dispersed on 2D sheet. It is suggested that no sub‐nanometer clusters were formed in the sample. Our method has two unique advantages for synthesizing SACs. First, the oxygen‐enriched precursor of sucrose is the key for constructing oxygen‐bonded SACs. Secondly, this NaCl templating method is low cost, highly scalable, and environmentally friendly because of high water solubility and recycling of NaCl.

The chemical state of Ni in Ni‐O‐G SACs was first probed with XPS (**Figure** **2**a), in which the Ni 2p_3/2_ XPS spectrum can be splitted as three peaks at 855.4, 858.6, and 862.3 eV respectively. According to previous literature, the peak at 862.3 eV can be associated with satellite peak, while the Ni 2p_3/2_ peaks located at 855.4 and 858.6 eV are higher than that of NiO (Figure S5a, Supporting Information), similar to the Ni species with higher valence state (i.e., Ni(III)).^33^, ^34^ In the O 1s spectrum (Figure S5b, Supporting Information), the peak at 531.9 and 530.6 eV can be assigned to the Ni—OH and Ni—O bonds,^35^ respectively, indicating diversified forms of oxygen coordinated to Ni SACs, similar to the FTIR results (Figure S5c, Supporting Information). Further, the spectra of X‐ray absorption near‐edge structure (XANES) and extended X‐ray absorption fine structure (EXAFS) at the Ni K‐edge were carried out to verify the possible bonding forms between nickel and the light elements in Ni‐O‐G SACs, in comparison with NiO and Ni foil. As seen from three curves of XANES (Figure 2b), the *E*_0_ values of Ni K‐edge (the first inflection point on the edge) followed the order of Ni‐O‐G SACs > NiO > Ni foil. Since higher *E*_0_ corresponds to higher oxidation state, it can be concluded that the valence state of Ni species in Ni‐O‐G SACs are higher than Ni (II), identical to the XPS result. Further, the coordination environment of Ni was elucidated by the Fourier‐transformed EXAFS (FT‐EXAFS, Figure 2c), in which Ni‐O‐G SACs showed only one notable peak at 1.6 Å, similar to the Ni‐O bond of NiO at ≈1.6 Å.^33^ Since nitrogen was free in Ni‐O‐G SACs, it confirmed that Ni atoms were mainly coordinated with oxygen. Moreover, no appearance of Ni—Ni coordination peak at 2.6 Å was detected, demonstrative of single atomic dispersion of oxygen bonded Ni in Ni‐O‐G SACs. Furthermore, EXAFS fitting was conducted to accurately quantitate the structural configuration of Ni‐O coordination in Ni‐O‐G SACs (Figure S6 and Table S1, Supporting Information). Simulation showed that the Ni SACs were neighborly coordinated with six oxygen atoms, revealing an oxygen saturated coordination structure and a slim probability of Ni—C bonds in Ni‐O‐G SACs. Based on the simulated result, we designed a six oxygen‐coordination model where Ni center atom was coordinated with four etherified O atoms and two OH groups as shown in the inset of Figure 2d, which was not only a thermal stable polyoxic structure but also well fitted with the FT‐EXAFS curve of Ni‐O‐G SACs (Figure 2d). In this model, the saturated oxygen coordination was favorable for the capture of electrons to form high valence state of Ni in SACs, resulting from oxygen‐enriched precursor of sucrose (C_12_H_22_O_11_) and high O/Ni ratio of >200 for sufficient oxygen coordination with Ni sites. Note that the two peaks at 3–3.5 Å from the simulated curve (red curve) in Figure 2d are the secondary peaks of highly crystallized graphene substrate not the Ni—Ni signals.^36^

**Figure 2:**
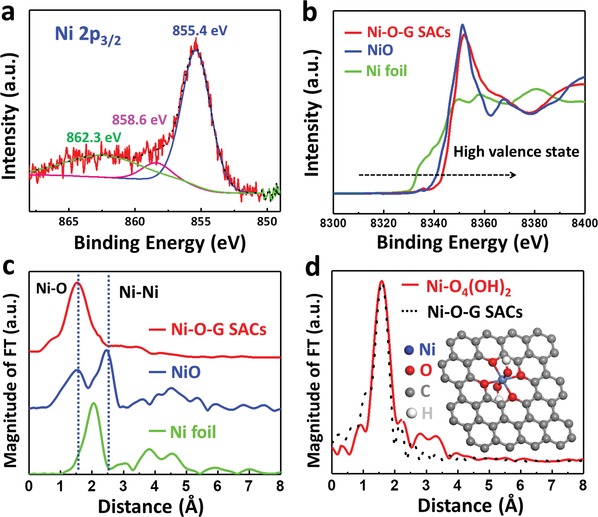
a) Ni 2p_3/2_ XPS spectrum of Ni‐O‐G SACs. b) Ni K‐edge XANES and c) FT‐EXAFS spectra of Ni‐O‐G SACs with references of NiO and Ni foil. d) The FT‐EXAFS curves of the proposed Ni−O_4_(OH)_2_ architecture (red line) and the measured Ni‐O‐G SACs (black line). Inset is the proposed model of Ni−O_4_(OH)_2_ architecture.

The catalytic activity of Ni‐O‐G SACs for OER was evaluated in alkaline electrolyte (Figure S7, Supporting Information). We first tuned the nickel single‐atom concentration to optimize the OER performance of Ni‐O‐G SACs. The result showed that the maximum activity of Ni‐O‐G SACs was obtained when the Ni content was ≈3.1 wt% (Figure S8, Supporting Information). To highlight the superiority of Ni‐O‐G SACs, we also compared the OER performance with the counterparts of bulk Ni‐O‐G (B Ni‐O‐G) without use of NaCl templates (Figure S9, Supporting Information), Ni‐N‐G SACs by replacing sucrose with nitrogen‐containing 2‐methimazole (Figure S10, Supporting Information), oxygen‐enriched graphene‐like carbon (O‐G) without addition of NiCl_2_ precursor (Figure S11, Supporting Information), NiO nanoparticles load on carbon cloth (NiO, Figure S12, Supporting Information), and commercial RuO_2_. Notably, the Ni‐O‐G SACs possessed extraordinary OER activity (**Figure** **3**a). To generate a current density of 10 mA cm^−2^, Ni‐O‐G SACs only required an overpotential of 224 mV, which is extremely lower than those of B Ni‐O‐G (420 mV), NiO (424 mV), Ni‐N‐G SACs (546 mV), O‐G (475 mV), and RuO_2_ (401 mV). Importantly, this value is the lowest of the reported pure Ni‐based catalysts, e.g., NiO film (540 mV),^37^ Ni/N doped graphene (397 mV),^38^ Ni_2_P/NiO*_x_* (286 mV),^26^ Ni_11_(HPO_3_)_8_(OH)_6_ (274 mV),^27^ Ni_3_Se_2_ (290 mV), and the state‐of‐the‐art OER SACs, such as Ni/defected graphene‐like nanosheets (270 mV) (Table S2, Supporting Information).^24^ Further, we performed the long‐term durability of Ni‐O‐G SACs at the overpotential of 240 mV, which showed a constant current density of 15 mA cm^−2^ for 14 h (Figure S7b, Supporting Information). It is confirmed that the current at low overpotential was resulting from the OER current, rather than the electro‐oxidation of Ni species.^37^ Moreover, the Tafel slope was fitted to be only 42 mV dec^−1^ for Ni‐O‐G SACs (Figure 3b), much lower than those of NiO (157 mV dec^−1^), B Ni‐O‐G (248mV dec^−1^), Ni‐N‐G SACs (328 mV dec^−1^), O‐G (253mV dec^−1^), and RuO_2_ (166 mV dec^−1^), respectively. It is noteworthy that, with such a smaller Tafel slope, the reaction kinetics was substantially activated, and Ni‐O‐G SACs achieved exceptionally higher current density with increased overpotential than others (Figure 3a). The intrinsic activity of Ni sites in Ni‐O‐G SACs was further confirmed by determining the turnover frequencies (TOF) at overpotential ranging from 200 to 400 mV (Figure 3c). In principle, a higher TOF values reflected the higher oxygen production rate of single Ni atomic site bonded with oxygen. Impressively, the TOF values of Ni‐O‐G SACs were 1.44 S^−1^ at 300 mV and 2.81 S^−1^ at 350 mV, exceeding the best values of OER catalysts reported, e.g., Cr^6+^/graphene (0.92 S^−1^ at 300 mV),^28^ CoFeW/Au (0.46 S^−1^ at 300 mV),^29^ NiFe‐layered double hydroxide (LDH)/carbon nanotube (CNT) (0.56 S^−1^ at 300 mV),^39^ NiFeOOH/Au (0.21 S^−1^ at 300 mV),^40^ 2D NiFe LDH(0.11 S^−1^ at 300 mV),^41^ and CoO_x_/Au (1.8 S^−1^ at 350 mV),^42^ respectively. Further, the long‐term durability of Ni‐O‐G SACs measured at 400 mV ovepotential showed exceptionally high initial current of 115 mA cm^−2^, without significant degradation after 50 h test (Figure 3d). HAADF‐STEM image verified the homogenous dispersion of Ni single atoms for Ni‐O‐G SACs even after the long‐term durability test (Figure 3d), revealing the highly robust stability of Ni‐O‐G SACs in OER.

**Figure 3:**
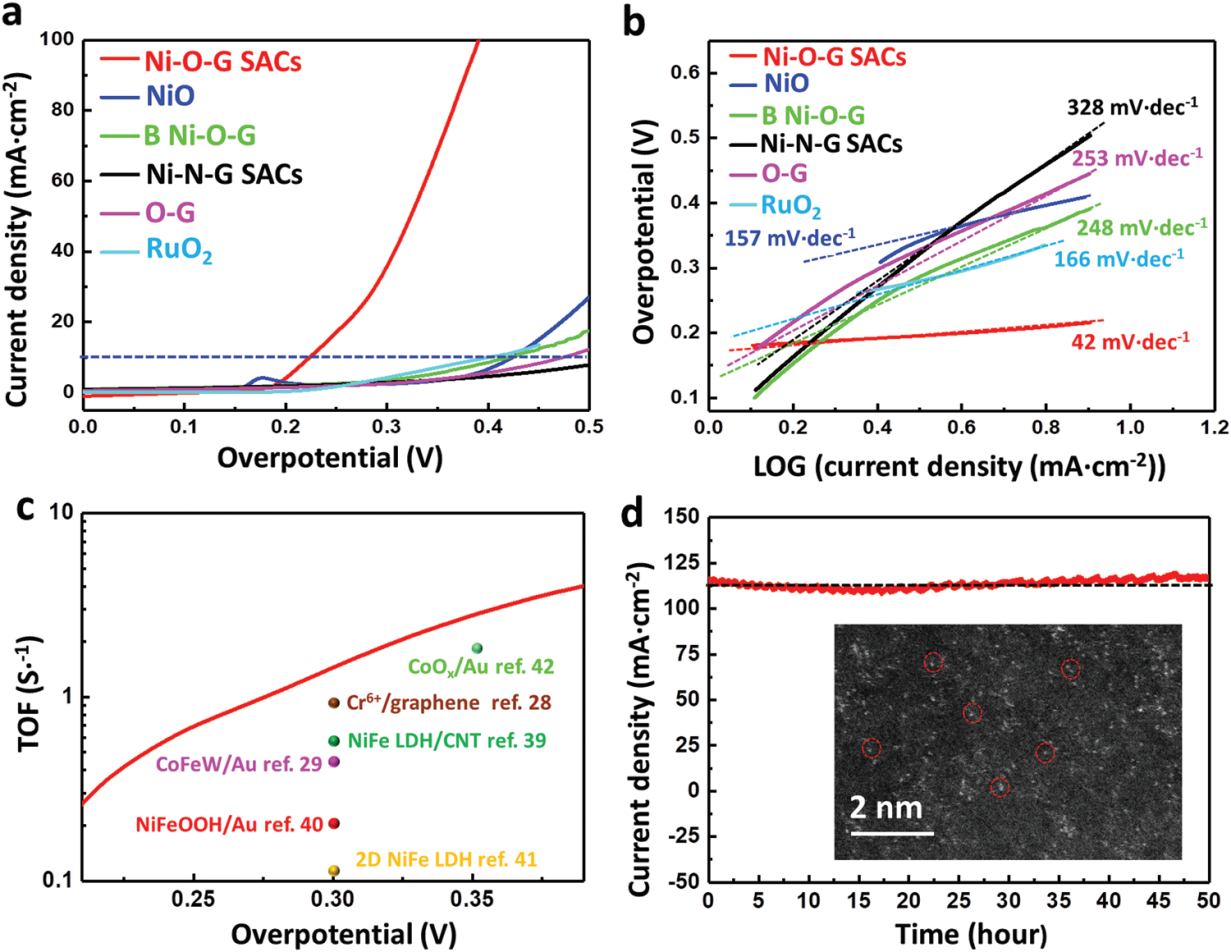
a) The OER current curves and b) corresponding Tafel plots of Ni‐O‐G SACs, NiO, B Ni‐O‐G, Ni‐N‐G SACs, O‐G, and RuO_2_ tested at 5 mV s^−1^ and 80% iR correction in 1 m KOH. c) The TOF curve of Ni‐O‐G SACs along with some recently reported OER catalysts. d) Chronoamperometric curve of Ni‐O‐G SACs obtained at constant overpotential of 400 mV in 1 m KOH, with 80% iR correction. Inset is the corresponding HAADF‐STEM image of Ni‐O‐G SACs after 50 h durability test. The single Ni atoms show bright dots marked with red circles.

To gain a fundamental understanding of high OER activity of Ni‐O‐G SACs, the first principle calculation was conducted to elucidate the state of single nickel (Ni) atoms in the optimal Ni‐O‐G geometry. The catalytic site was assumed as the Ni atom in Ni‐O_4_(OH)_2_ configuration (**Figure** **4**a) and the calculated structure was based on the model reported by Jiang et al.^43^ As the disparity in the electronegativity of Ni (1.8) and O (3.5),^44^ the electrons prefer to accumulate on the side of oxygen atoms. Thus, the electrostatic potential (ESP) of nickel atoms inclined to be more positive, resulting in the more positively charged nickel atoms with high theoretical oxidation state (+2.34, Figure 4b), as confirmed by XPS and EXFAS results.^45^ For the OER process, it usually involves four adsorption/desorption steps (Figure 4c): i) OH^−^ is adsorbed on catalytic sites and converted as *OH; ii) *OH is formed as *O; iii) *O bonding to OH^−^ forms *OOH; and iv) *OOH is dissociated into O_2_ which is released from the catalysts.^46^ The profile of free energy variation (Δ*G*) was calculated with an external potential of 1.23 V (Figure 4d). Further, we create the model of NiO nanoparticles (Figure S13, Supporting Information) and single Ni atoms bond to N elements on graphene (Ni‐N‐G SACs SACs; Figure S14, Supporting Information) to calculate their free energy variations in OER processes for comparison. Correspondingly, the oxidation state of Ni sites for NiO and Ni‐N‐G SACs is calculated to be +1.84 and +1.57, respectively, much lower than that of Ni‐O‐G SACs (+2.37). As a consequence, the ability of individual nickel sites for adsorbing oxygen groups in Ni‐O‐G SACs is higher than that in NiO and Ni‐N‐G SACs. It is revealed that the formation of *O (Δ*G*_2_) is the rate‐determining step for the three models. Further, the Ni site in Ni‐O‐G SACs, NiO and Ni‐N‐G SACs showed an energy barrier of 0.48, 0.53, and 1.09 eV, corresponding to the theoretical overpotential of 0.48, 0.53, and 1.09 V, respectively. Therefore, this strategy by constructing oxygen bonding with single Ni atoms could extraordinarily boost the OER activity and durability.

**Figure 4:**
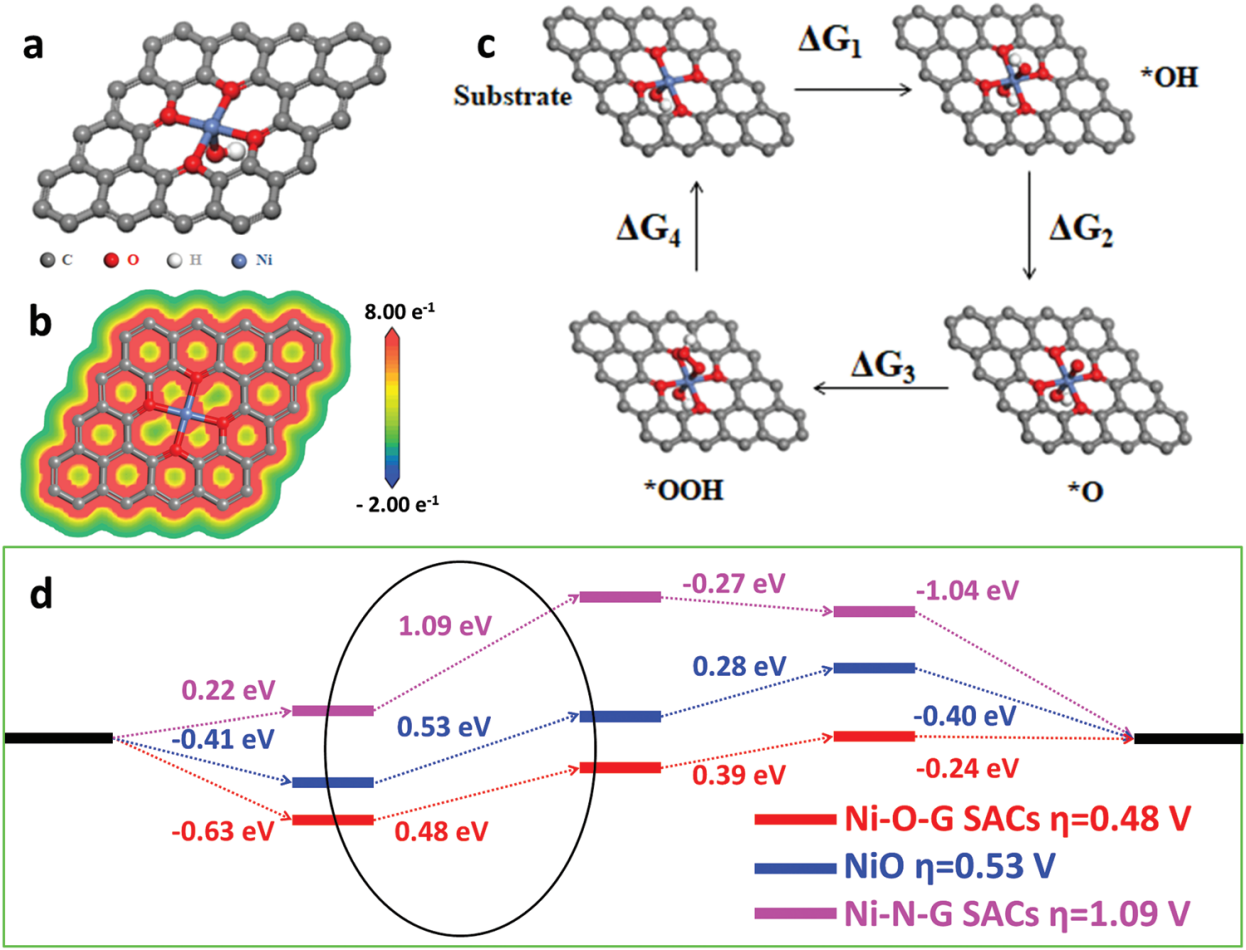
a) Optimized geometric model of Ni sites in Ni‐O‐G SACs structure. b) The corresponding map of the DFT ESP surfaces of Ni‐O‐G SACs structure. Blue color indicates positive charges, and red color indicates negative charge. c) Schemeric of oxygen production pathways on the Ni site within Ni‐O‐G SACs geometry. d) The free‐energy diagrams of OER pathways and OER theoretical overpotential of the Ni‐O‐G SACs structure (red), Ni‐N‐G SACs (pink), and NiO nanoparticles (blue).

In summary, we have developed novel‐type Ni‐O‐G SACs, with 3D porous framework, 2D ultrathin sheet structure, uniform distribution of individual Ni atoms, appropriate Ni—O coordination, and high valence state of >Ni(II) species, were demonstrated for remarkably activating OER with superior activity and durability. The Ni‐O‐G SACs showed a very low overpotential of 224 mV at the current density of 10 mA cm^−2^ and 42 mV dec^−1^ Tafel slope, 1.44 and 2.81 S^−1^ of oxygen production TOF at the overpotential of 300 and 350 mV, respectively, far outperforming the state‐of‐the‐art OER SACs. The computational simulation uncovered that the Ni—O bonding in the Ni‐O_4_(OH)_2_ configuration, with high oxidation state of single Ni atoms resulted in low OER overpotential and reduced Gibbs‐free energy for Ni‐O‐G SACs. We believe that this work will pave the way for creating highly active and stable SACs through the chemical coordination of single metal atoms and oxygen sites of 2D substrate for many electrocatalytic reactions and systems.

## Experimental Section

*Preparation of Ni‐O‐G SACs*: Typically, 300 mg sucrose, 10 mg NiCl_2_·6H_2_O and 20 mg H_2_SO_4_ were dispersed in 10 mL deionized water, followed by addition of 3 g NaCl while stirring. The mixture was frozen to the solid state by liquid nitrogen several minutes and the ice cube was freeze‐dried for 2 d to remove water. Freeze‐drying condition is −50 °C and 20 Pa. Afterwards, the freeze‐dried sample was heated in a muffle furnace at 100 °C for 5 h and then the sample was annealed at argon atmosphere at 700 °C for 2 h. The Ni‐O‐G SACs were obtained by washing the annealed sample to remove the NaCl templates with deionized water. To determine the content of nickel accurately, 100 mg Ni‐O‐G SACs was annealed in air at 1000 °C for 1 h to remove carbon, and only the residual substances of NiO were left. The quantity of the resulting NiO was 4.1 mg, thus the Ni content was calculated to be 3.1 mg. Therefore, the Ni content in this Ni‐O‐G SAC sample is ≈3.1 wt%.

The Ni content in Ni‐O‐G SAC samples were tuned from 0.85 and 2.24 wt% to 3.1 wt% by changing the adding quantity from 3 and 7 to 10 mg of NiCl_2_·6H_2_O, respectively, while other experimental procedures were kept the same as the Ni‐O‐G SACs with 3.1 wt% loading of Ni single atoms.

For comparison, the B Ni‐O‐G (Ni 3.1 wt%) was prepared by the same procedure of Ni‐O‐G SACs, only without adding NaCl. Similarly, the Ni‐N‐G SACs was synthesized by replacing 300 mg sucrose with 800 mg 2‐methimazole, and the Ni amount was ≈3.5 wt%. Also, the O‐G was prepared using the same synthesis of Ni‐O‐G SACs without addition of NiCl_2_·6H_2_O.

*Preparation of NiO*: First, the commercial carbon cloth was cut into 1 × 2 cm, followed by washing with deionized water and ethanol. Then, it was immersed in mixture of 2 mL ethanol and 3 mL water containing 6 mg Ni(NO_3_)_2_·6H_2_O for 10 min. Afterwards, the carbon cloth with the adsorbed Ni^2+^ions was dried at 60 °C without washing, and annealed at 250 °C for 3 h. Finally, NiO was obtained on carbon cloth.

*Materials Characterizations*: The prepared samples were studied by powder XRD, which was performed on a Bede D1 system operated at 20 kV and 30 mA with Cu Kα radiation (λ = 1.5406 Å). SEM images were tested with the JSM‐7900F (JEOL, Japan). High‐resolution transmission electron microscopy (HRTEM, JEOL 2100) was used to identify the morphology and crystal structure of the nanostructures. EDS mapping were imaged with FEI (F30). The aberration‐corrected HAADF‐STEM was detected by FEI Titan Cube Themis G2 300, 300 kV equipped with two spherical aberration correctors. XPS was recorded on a Thermo ESCALAB‐250 spectrometer using a monochromatic Al Kα radiation source (1486.6 eV). The binding energies determined by XPS were corrected by reference to the adventitious carbon peak (284.6 eV) for each sample. Raman spectra were recorded on a HORIBA Raman spectrometer, with an excitation laser wavelength of 532 nm. The FTIR was tested by HYPERION 3000 (Bruker Optics). The ex situ Ni K‐edge EXAFS data were collected on the beamline BL14W1 at Shanghai Synchrotron Radiation facility (SSRF). All samples were prepared by placing a small amount of homogenized (via agate mortar and pestle) powder on 3M tape. The IFEFFIT software was used to calibrate the energy scale, to correct the background signal and to normalize the intensity. The spectra were normalized with respect to the edge height after subtracting the pre‐edge and post‐edge backgrounds using Athena software. To extract EXAFS oscillations, the background was removed in k‐space using a five‐domain cubic spline. The resulting k‐space data, k3χ(k), was then FT.

*Electrochemical Measurements*: The working electrode was fabricated as follows: first, commercial carbon cloth (with size of 1 × 2 or 1 × 1 cm) was washed with deionized water and ethanol. Secondly, 2.0 mg catalyst was dispersed in 1.0 mL ethanol, and thus 0.01 mL Nafion solution (5.0 wt%) was added, followed by 1.0 h sonication to form the homogeneous suspension. Afterwards, 100 µL (or 50 µL) of the suspension was pipetted onto the carbon cloth with 2 cm^2^ (or 1 cm^2^). After the solvent evaporation for 30 min in air, the working electrode with catalyst loading of 0.1 mg cm^−2^ was obtained for OER measurements.

The electrochemical measurements were carried out at the room temperature, using an electrochemical workstation (CHI 760E) with a typical three‐electrode system, in which the carbon cloth with the loaded catalyst was used as working electrode, a Pt sheet (2 × 2 cm) was as counter electrode and an Ag/AgCl electrode as reference electrode, respectively. All potentials measured were calibrated to the reversible hydrogen electrode (RHE), using the following equation:

(1) $$E_{\text{RHE}}=E_{\text{Ag/AgCl}}+0.197\;\;\text{V}+0.059*\text{pH}$$

(2) $$\text{and}\;\text{the}\;\text{overpotential}=E_{\text{RHE}}-1.23\;\;\text{V}$$

For OER tests, first, the working electrodes were scanned for several potential cycles until the signals were stabilized. Linear scan voltammetry (LSV) measurements were performed at a scan rate of 5 mV s^−1^. Tafel slopes were calculated based on the LSV curves by plotting overpotential against log (current density). The impedance was very consistent at multiple potential points (covering both non‐OER and OER conditions). All polarization curves were corrected with 80% iR correction. The electrochemical impedance spectroscopy was carried out in a potentiostatic mode at 1.26 V versus RHE (overpotential of 93 mV), applying a sinusoidal voltage with an amplitude of 10 mV and a scanning frequency from 1 m to 0.01 Hz.

The electrochemically active surface areas were measured on the same working electrodes. The double‐layer capacitance *C*_dl_ was extracted from LSV curves in non‐faradaic and OER potential regions (0.25–0.35 V versus Ag/AgCl, 1 m KOH) at the scan rates of 2, 4, 6, 8,10 and 12 mV s^−1^.The double‐layer capacitance (*C*_dl_) was estimated by plotting the Δ*j* (*j*_anodic_ − *j*_cathodic_) at 0.30 V versus Ag/AgCl against the scan rate. The linear slope is twice of the double‐layer capacitance *C*_dl_.

The TOF value was calculated from the equation: κ = *I*/(*n* × *F* × 4). *I* represents the Coulomb number of electron quantity in 1 s. *n* represents the mole number of Ni atoms loaded in electrode. *F* was the Faraday constant (96 485).

*First‐Principle Calculations*: The graphene‐Ni complexes (Ni‐O‐G SACs and Ni‐N‐G SACs) were constructed based on a 6 × 6 × 1 graphene supercell with two vacuum layers of 30 Å on both the top and down sides of the graphene layer, the NiO nanoparticle model was based on a 2 × 2 × 2 supercell, one hydroxyl group was pre‐coordinated with Ni atom. For the OER processes, the calculations were performed by using the Vienna ab initio simulation package, based on the spin‐polarized DFT within the generalized gradient approximation and Perdew–Burke–Ernzerhof functional. The electron wave functions are expanded by using a plane wave basis set with a cut‐off energy of 400.0 eV. In the structure relaxations, the atomic geometries are fully optimized until the threshold forces less than 0.005 eV Å^−1^. Four adsorption/desorption steps are usually considered in conventional single site OER mechanism:

(3) $${\text{H}}_{2}\text{O}+*\to *\text{OH}+\text{H}\;\Delta G_{1}$$

(4) $$*\text{OH}\to *\text{O}+\text{H}\;\Delta G_{2}$$

(5) $$*\text{O}+{\text{H}}_{2}\text{O}\to *\text{OOH}+\text{H}\;\Delta G_{3}$$

(6) $$*\text{OOH}\to {\text{O}}_{2}+*+\text{H}\;\Delta G_{4}$$

where * presents the adsorption site that is usually on the top of the active atoms, Δ*G*_1_ ≈ Δ*G*_4_ are the change in Gibbs‐free energy for each step.

## Conflict of Interest

The authors declare no conflict of interest.

## Supporting information

Supporting Information
