# Supplementary material for: High‐Valence Nickel Single‐Atom Catalysts Coordinated to Oxygen Sites for Extraordinarily Activating Oxygen Evolution Reaction
Source: Adv Sci (Weinh). 2020 Jan 20;7(5):1903089. doi: 10.1002/advs.201903089 (PMC7055577; doi:10.1002/advs.201903089)
Supplement: Supplementary file 1 — Supporting Information [file ADVS-7-1903089-s001.pdf]

Copyright WILEY-VCH Verlag GmbH & Co. KGaA, 69469 Weinheim, Germany, 2020.

Supporting Information

for *Adv. Sci.,* DOI: 10.1002/advs.201903089

High-Valence Nickel Single-Atom Catalysts Coordinated to Oxygen Sites for Extraordinarily Activating Oxygen Evolution Reaction

Yaguang Li, Zhong-Shuai Wu, Pengfei Lu, Xiao Wang, Wei Liu, Zhibo Liu, Jingyuan Ma, Wencai Ren, Zheng Jiang,* and Xinhe Bao*

((Supporting Information can be included here using this template))

Copyright WILEY-VCH Verlag GmbH & Co. KGaA, 69469 Weinheim, Germany, 2019.

Supporting Information

High-Valence Nickel Single-Atom Catalysts Coordinated to Oxygen Sites for Extraordinarily Activating Oxygen Evolution Reaction

Yaguang Li, Zhong-Shuai Wu, Pengfei Lu, Xiao Wang, Wei Liu, Zhibo Liu, Jingyuan Ma, Wencai Ren, Zheng Jiang,* Xinhe Bao*

Table S1. EXAFS fitting parameters of Ni with reference samples extracted from the Ni K-edge

sample	Path	CN	$\sigma^2(10^{-3}$ Å$^2)$	R	ΔE_0(eV)
Ni foil	Ni-Ni	12	5.9±0.4	2.48±0.03	7.5
NiO	Ni-O	6	7.6±0.3	2.08±0.02	-3.9
	Ni-Ni	12	7.2±1.0	2.94±0.03	-4.6
$Ni(OH)_2$	Ni-O	6	6.4±1.0	2.06±0.01	-4.4
	Ni-Ni	6	7.4±1.0	3.13±0.01	-2.3
Ni-O-G SACs	Ni-O	6.0±1.0	3.6±1.2	2.05±0.03	-6.1

Note: The amplitude reduction factor S_0^2 is 0.78, and the Fourier transformation of the k^3-weighted EXAFS oscillations, $k^3 \cdot \chi(k)$, from k space to R space, was performed over a range of 3.1–13.5 Å^{-1}.

Table S2. OER performance comparison of Ni-O-G SACs and the state-of-the-art Ni based OER electrocatalysts

Catalyst	Support	Overpotential at 10 mA·cm^{-2} (mV)	Electrolyte	Refs
S Ni/defected graphene	glassy carbon	270	1 M KOH	[1]
NiO film (8 nm thick)	FTO	540	0.1 M KOH	[2]
Ni/N doped graphene	glassy carbon	397	0.1 M KOH	[3]
Ni_2P/NiO_x	glassy carbon	286	1 M KOH	[4]
$Ni_{11}(HPO_3)_8(OH)_6$	FTO	274	1 M KOH	[5]
Ni_3Se_2	glassy carbon	290	1 M KOH	[6]
NiN_4C_4	glassy carbon	331	1 M KOH	[7]
Ni-O-G SACs	carbon cloth	224	1 M KOH	This work

Table S3. The zero point energies and entropic corrections of oxygenates at 298 K

Species	NiO		NiN_4-graphene		NiO_4OH-graphene	
	ZPE	TS	ZPE	TS	ZPE	TS
**O***	0.317	0.00	0.041	0.00	0.041	0.00
**OH***	0.556	0.00	0.317	0.00	0.327	0.00
**OOH***	0.676	0.00	0.422	0.00	0.403	0.00
**O_2**	0.10	0.64	0.10	0.64	0.10	0.64
**H_2**	0.34	0.41	0.34	0.41	0.34	0.41
**H_2O**	0.57	0.67	0.57	0.67	0.57	0.67

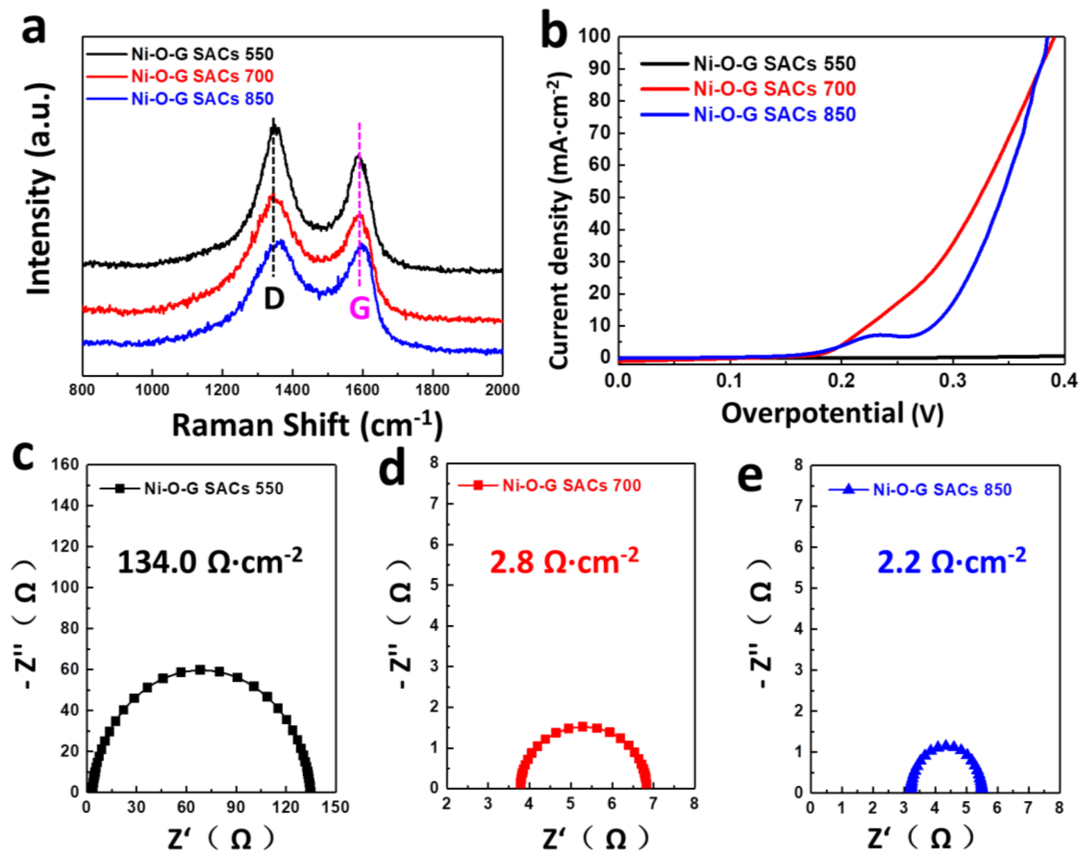

Figure S1. (a) Raman spectra, and (b) OER current curves tested at 5 mV·s^{-1} and 80% *iR* correction in 1 M KOH of Ni-O-G SACs 550, Ni-O-G SACs 700, and Ni-O-G SACs 850, respectively. (c-e) Nyquist plots of (c) Ni-O-G SACs 550, (d) Ni-O-G SACs 700 and (e) Ni-O-G SACs 850. It was revealed that the charge transfer resistance of Ni-O-G SACs 550, Ni-O-G SACs 700, and Ni-O-G SACs 850 were 134.0, 2.8 and 2.2 Ω·cm^{-2}, respectively.

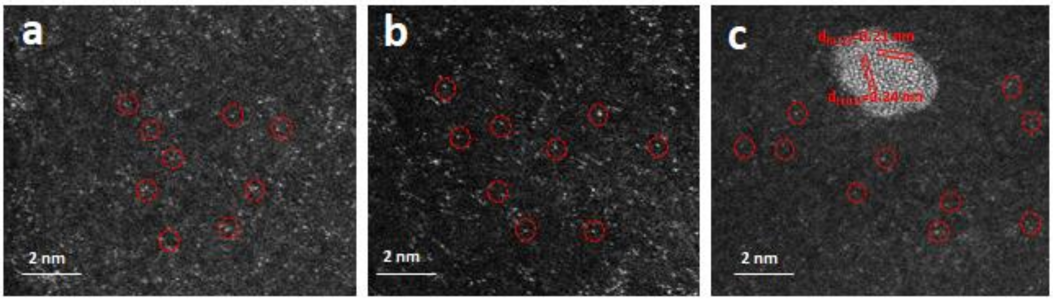

Figure S2. (a, b, c) Atomic-scale HAADF-STEM images of (a) Ni-O-G SACs 550, (b) Ni-O-G SACs 700 showing the uniform Ni single atoms distribution, and (c) Ni-O-G SACs 850 showing the co-existed appearance of NiO (PDF# 44-1159) nanoparticles (3~5 nm) and Ni single atoms, as indicated by red circles.

To investigate the effect the I_D/I_G ratio on OER performance, we have prepared the Ni-O-G SACs samples annealed at different temperatures of 550 °C, 700 °C, and 850 °C, which were denoted as Ni-O-G SACs 550, Ni-O-G SACs 700, and Ni-O-G SACs 850, respectively. The I_D/I_G ratio was gradually reduced with increasing the annealing temperature, for example, 1.89 for Ni-O-G SACs 550, 1.39 for Ni-O-G SACs 700, and 1.07 Ni-O-G SACs 850 (Figure S1a). The catalytic activity of three Ni-O-G SACs samples for OER was evaluated using a standard three-electrode system in 1 M KOH electrolyte. It was observed that the Ni-O-G SACs 550 showed neglectable OER activity (Figure S1b) possibly caused by low electrical conductivity derived from large amount of defects, as confirmed by EIS measurement, in which Ni-O-G SACs 550 showed larger charge transfer resistance of 130 $\Omega\cdot cm^{-2}$ (Figure S1c) than those of Ni-O-G SACs 700 (2.8 $\Omega\cdot cm^{-2}$, Figure S1d) and Ni-O-G SACs 850 (2.2 $\Omega\cdot cm^{-2}$, Figure S1e). Therefore, the charge transfer resistance is reduced with the decreased I_D/I_G ratio.

Further, atomic-scale HAADF-STEM images of both Ni-O-G SACs 550 and Ni-O-G SACs 700 displayed the bright tiny dots of uniform single Ni atoms (Figure S2a, b), while Ni-O-G SACs 850 exhibited the co-existance of NiO nanoparticles (3~5 nm) and Ni single atoms. As a result, the ability to anchor a Ni single atom is greatly weakens with the decreased I_D/I_G ratio. As confirmed, the Ni-O-G SACs 700 showed a larger overpotential of 224 mV, in comparison with Ni-O-G SACs 850 (274 mV, Figure S1b), due to the presence of

NiO nanoparticles. Therefore, we consider that an appropriate I_D/I_G ratio, optimized by tuning temperature (*e.g.*, 700 °C), is critical for the formation of mono-dispersed Ni SACs bonded to oxygen sites of graphene-like sheets.

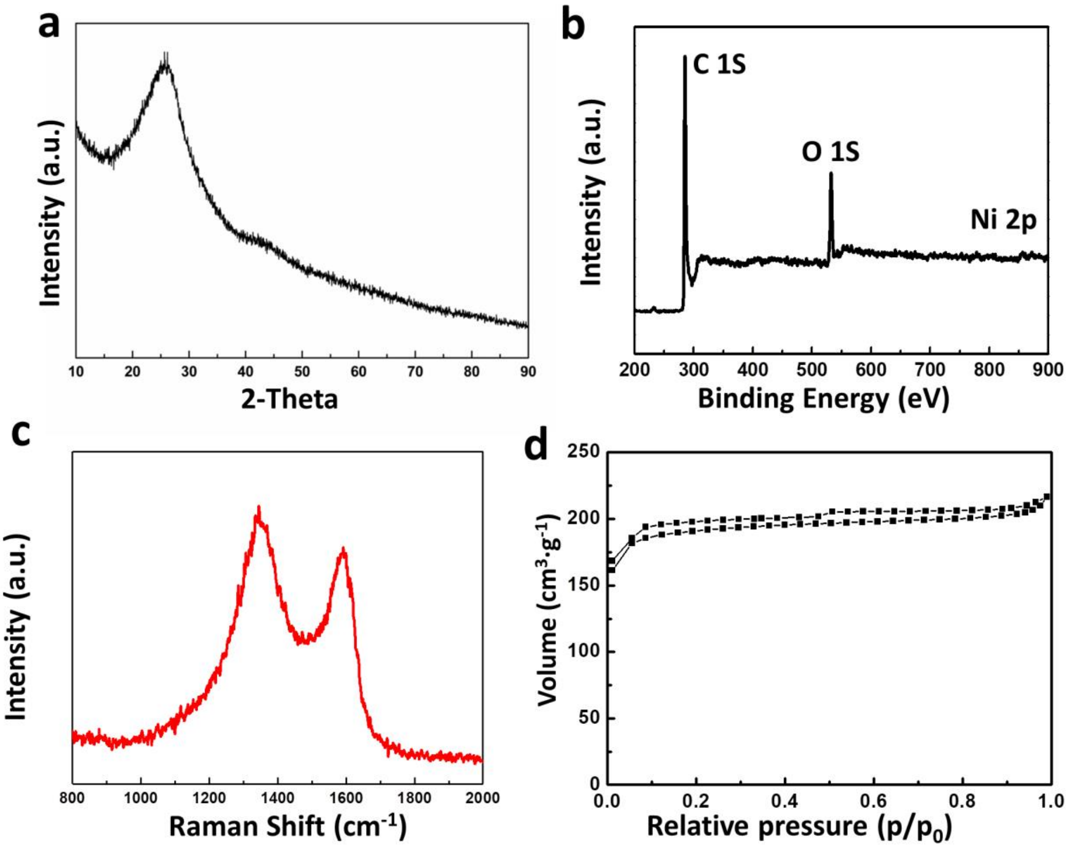

Figure S3. (a) XRD pattern, (b) full XPS spectrum, (c) Raman spectrum, (d) nitrogen adsorption and desorption isotherm of Ni-O-G SACs.

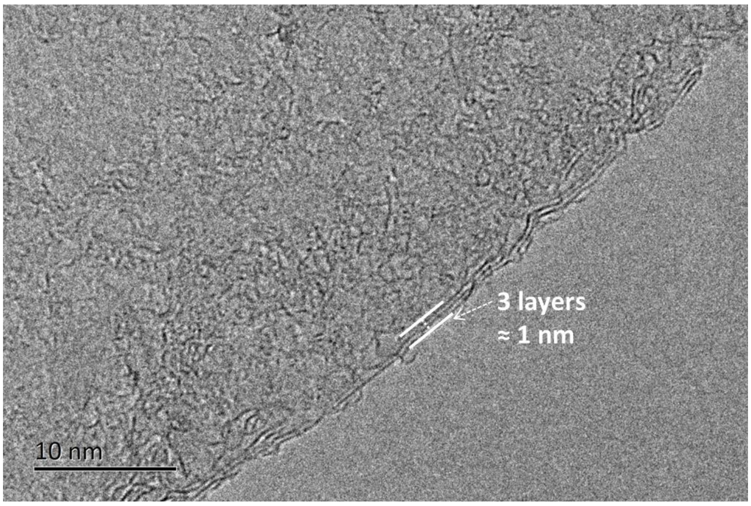

Figure S4. HRTEM image of Ni-O-G SACs, showing smooth surface and the number of ≤ 3 layers.

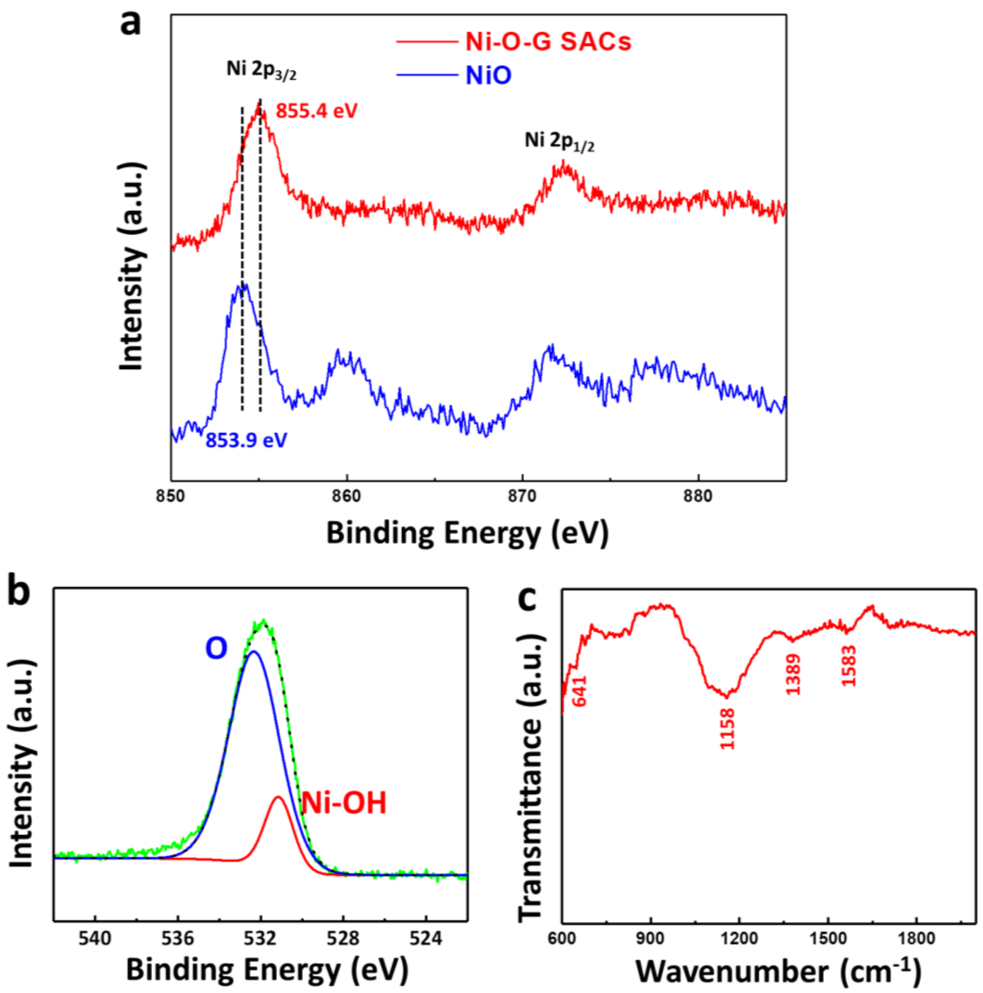

Figure S5. (a) Ni 2p XPS spectra of Ni-O-G SACs (red) and NiO (blue). (b) O 1s XPS and (c) FTIR spectra of Ni-O-G SACs. The band located at 1158 cm^{-1} is probably related to the presence of carbonate ions derived from the adsorption of atmospheric CO_2. Moreover, the band at 1389 cm^{-1} and 1583 cm^{-1} can be attributed to the anions, whereas the band around 641 cm^{-1} is ascribed to the δ_{OH} vibrations.[8]

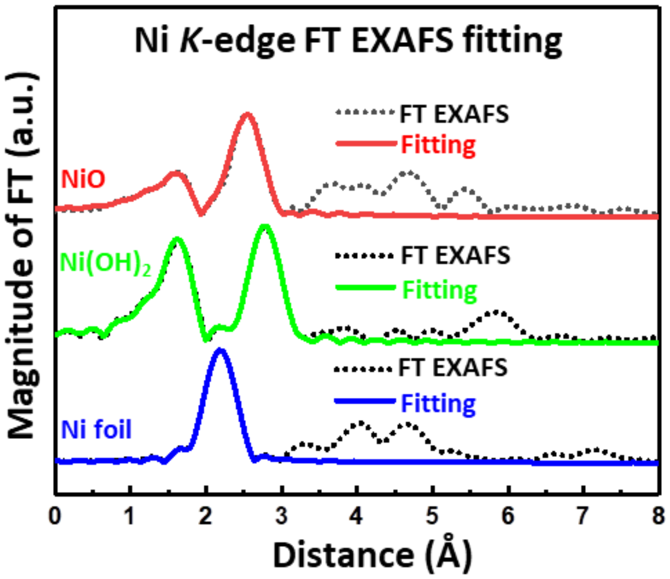

Figure S6. FT-EXAFS fitting of Ni K-edge in Ni-O-G SACs with references.

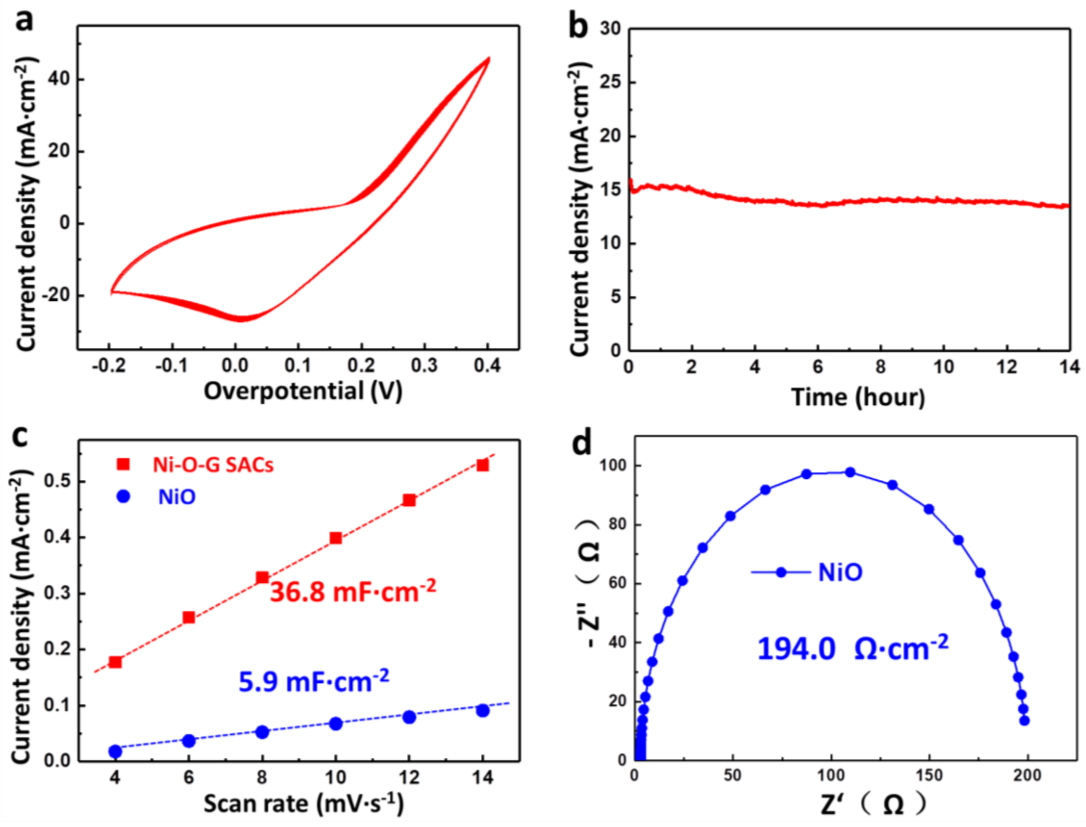

Figure S7. (a) Cyclic voltammogram of Ni-O-G SACs with 30 cycles in 1 M KOH, without *iR* correction. (b) Chronoamperometric curve of Ni-O-G SACs obtained at the overpotential of 240 mV in 1 M KOH, with 80% *iR* correction. (c) Charging current density differences versus scan rate of Ni-O-G SACs and NiO. (d) Nyquist plots of NiO. It is revealed that the ECSA and charge transfer resistance of Ni-O-G SACs were 36.8 mF·cm^{-2} (Figure S7c) and 2.8 Ω·cm^{-2} (Figure S1d) respectively, both of which are much lower than those of NiO (5.9 mF cm^{-2}, 194.0 Ω·cm^{-2}). It can be explained that high active surface area and conductivity of Ni-O-G SACs electrodes ensures large number of catalytic sites and efficient charge transfer between active sites and electrode substrate to achieve large current densities at low overpotential.

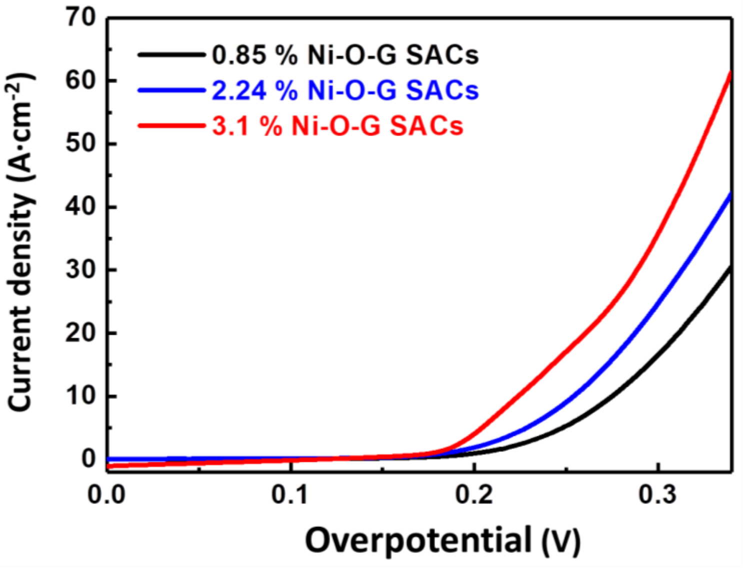

Figure S8. The OER curves of Ni-O-G SACs with different Ni content, tested at scan rate of 5 mV·s^{-1}, with 80% *iR* correction.

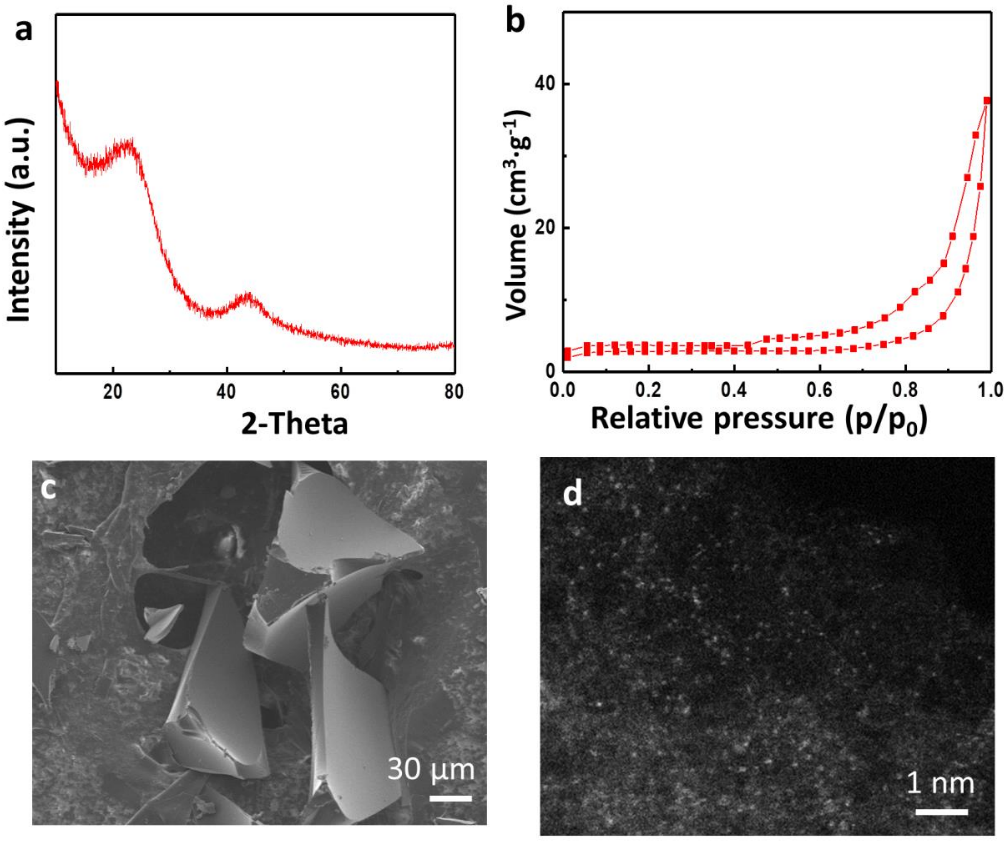

Figure S9. (a) XRD pattern, (b) nitrogen adsorption and desorption isotherm, (c) SEM image and (d) HAADF-STEM image of B Ni-O-G. XRD pattern presented two broad diffraction peaks at 23.2 ^{o} and 43.6 ^{o}, derived from pure carbon materials.[9] Besides this, no any other peaks of Ni metals, metal oxides, metal carbides were observed, indicative of homogenous dispersion of individual Ni species.[10]

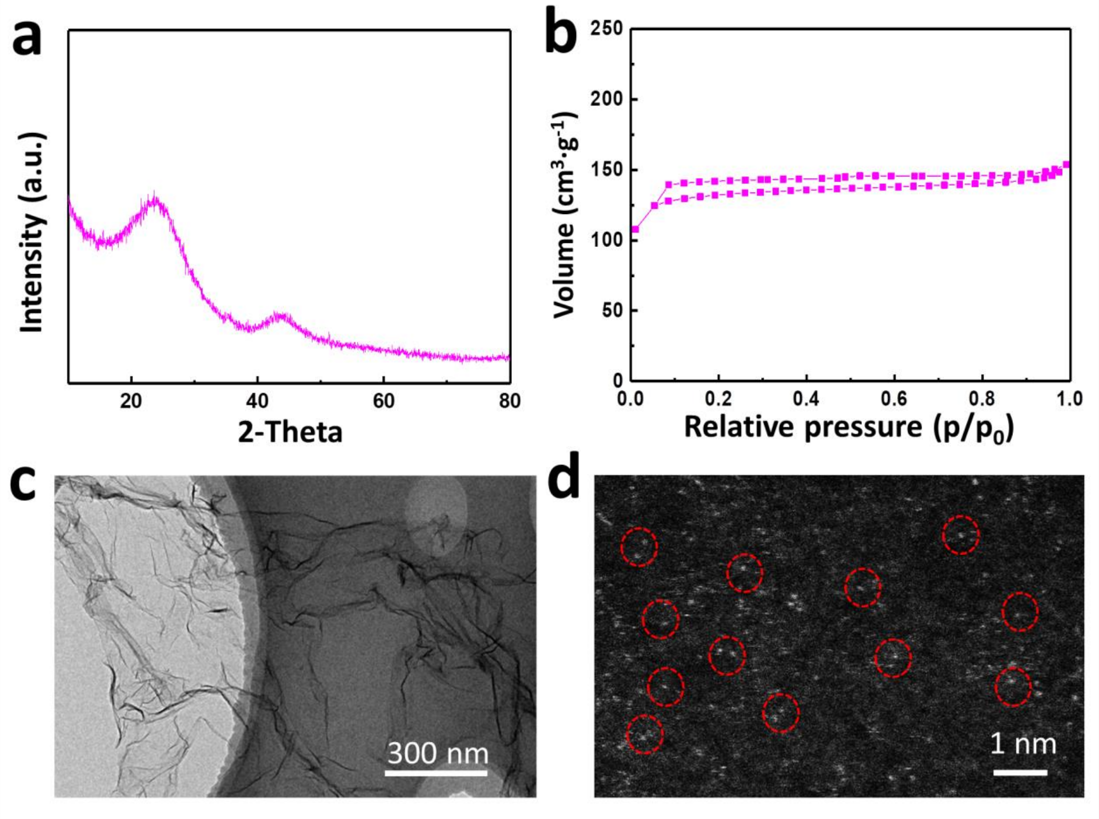

Figure S10. (a) XRD pattern, (b) nitrogen adsorption and desorption isotherm, (c) TEM image and (d) HAADF-STEM image of Ni-N-G SACs. The single Ni atoms show bright dots marked with red circles. XRD pattern presented two broad diffraction peaks at 23.2 ^{o} and 43.6 ^{o}, indicative of the nature of carbon.[9] Besides this, no other peaks of Ni metals, metal oxides and metal carbides were appeared (Figure S10a), indicative of uniform dispersion of individual Ni species.[10] Metal nanoparticles were absent in TEM image of Ni-N-G nanosheets (Figure S10c). Further, the Ni single atoms were confirmed by the atomic-scale HAADF-STEM image (Figure S10d).

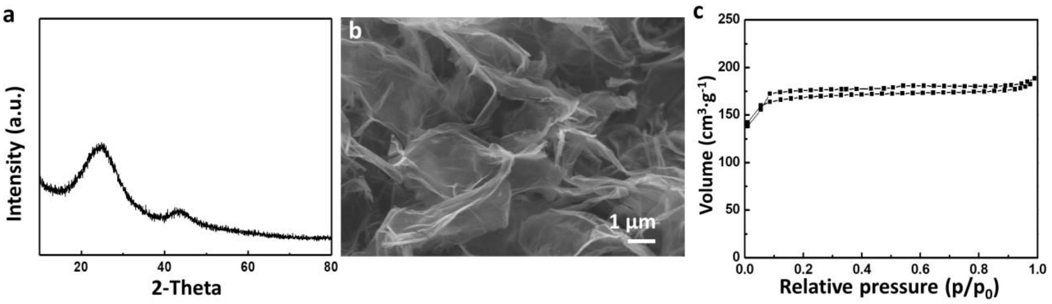

Figure S11. (a) XRD pattern, (b) SEM image and (c) nitrogen adsorption and desorption isotherm of O-G.

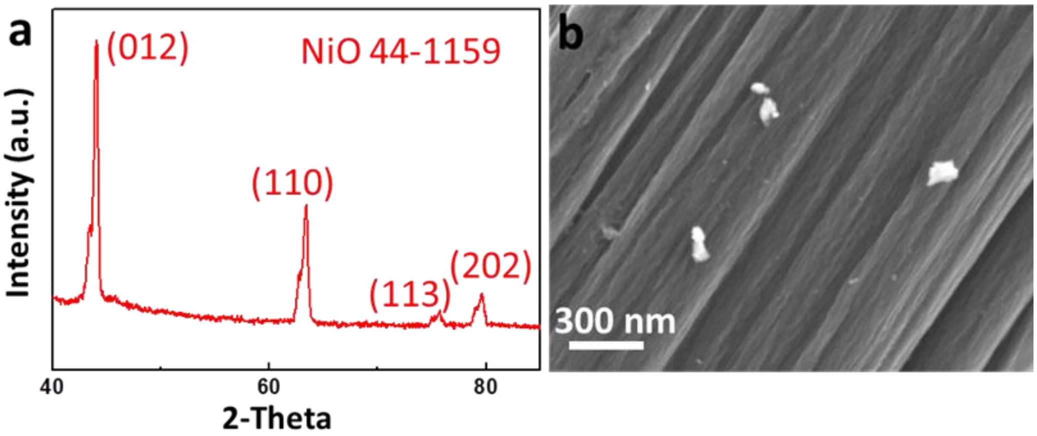

Figure S12. (a) XRD pattern and (b) SEM image of NiO on carbon cloth.

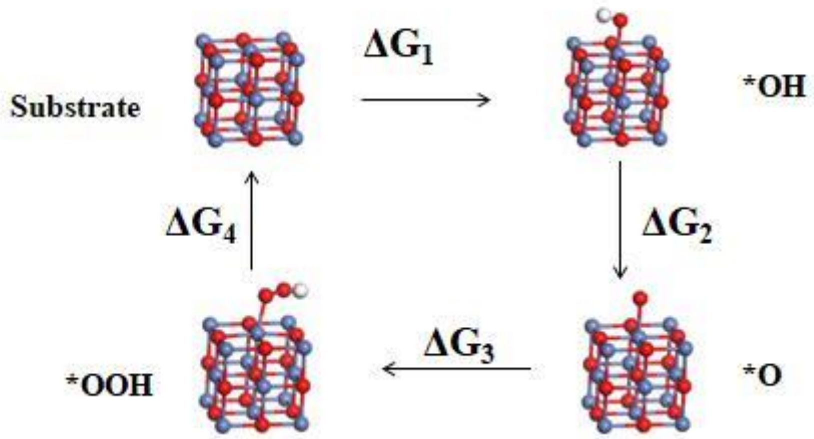

Figure S13. Schemeric of oxygen production pathways on the Ni site within NiO nanoparticles.

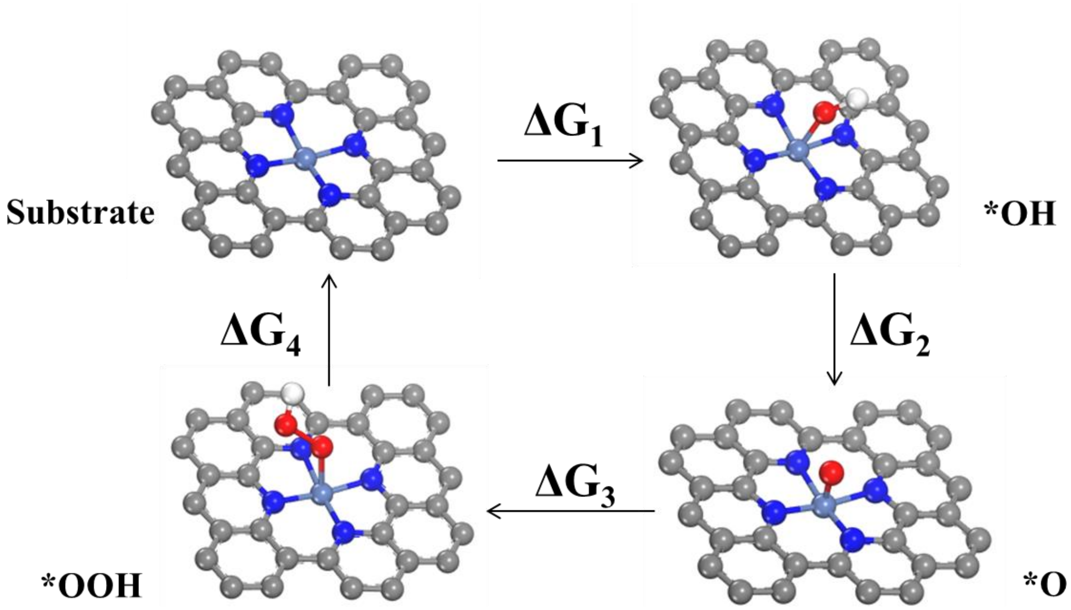

Figure S14. Schemeric of oxygen production pathways on the Ni site within Ni-N-G SACs in NiN_4 model. The structure is refereed by the literature reported by Li *et al*.[9]

References

[1] L. Zhang, Y. Jia, G. Gao, X. Yan, N. Chen, J. Chen, M. T. Soo, B. Wood, D. Yang, A. Du, X. Yao, *Chem* **2018**, 4, 285.

[2] K. L. Nardi, N. Yang, C. F. Dickens, A. L. Strickler, S. F. Bent, *Adv. Energy. Mater.* **2015**, 5, 1500412.

[3] S. Chen, J. Duan, J. Ran, M. Jaroniec, S. Z. Qiao, *Energy Environ. Sci.* **2013**, 6, 3693.

[4] L.-A. Stern, L. Feng, F. Song, X. Hu, *Energy Environ. Sci.* **2015**, 8, 2347.

[5] P. W. Menezes, C. Panda, S. Loos, F. Bunschei-Bruns, C. Walter, M. Schwarze, X. Deng, H. Dau, M. Driess, *Energy Environ. Sci.* **2018**, 11, 1287.

[6] A. T. Swesi, J. Masud, M. Nath, *Energy Environ. Sci.* **2016**, 9, 1771.

[7] H. Fei, J. Dong, Y. Feng, C. S. Allen, C. Wan, B. Volosskiy, M. Li, Z. Zhao, Y. Wang, H. Sun, P. An, W. Chen, Z. Guo, C. Lee, D. Chen, I. Shakir, M. Liu, T. Hu, Y. Li, A. I. Kirkland, X. Duan, Y. Huang, *Nat. Catalysis* **2018**, 1, 63.

[8] J. Yan, Z. Fan, W. Sun, G. Ning, T. Wei, Q. Zhang, R. Zhang, L. Zhi, F. Wei, *Adv. Funct. Mater.* **2012**, 22, 2632.

[9] C. Zhao, X. Dai, T. Yao, W. Chen, X. Wang, J. Wang, J. Yang, S. Wei, Y. Wu, Y. Li, *J. Am. Chem. Soc.* **2017**, 139, 8078.

[10] W. Liu, Y. Chen, H. Qi, L. Zhang, W. Yan, X. Liu, X. Yang, S. Miao, W. Wang, C. Liu, A. Wang, J. Li, T. Zhang, *Angew. Chem. Int. Ed.* **2018**, 57, 7071.
